# Development of the lexicon, trained panel validation and sensory profiling of new ready-to-eat plant-based " *meatballs*" in tomato sauce

**DOI:** 10.12688/openreseurope.15360.3

**Published:** 2024-02-13

**Authors:** Clara Talens, Maider Lago, Eder Illanes, Ana Baranda, Mónica Ibargüen, Elena Santa Cruz

**Affiliations:** 1 AZTI, Food Research, Basque Research and Technology Alliance (BRTA), Derio, Bizkaia, 48160, Spain

**Keywords:** healthy nutritional habits, sensory analysis, trained panel, ready-to-eat meals, plant-based, meat analogues

## Abstract

**Background:**

Providing educational content for children and parents can promote healthy nutritional habits. During the TITAN project, a pilot digital contest where participants have to developed ready-to-eat meatballs in sauce, using only plant-based ingredients, will be tested. The objective of this study was to develop the lexicon needed to objectively assess the sensory profile of this product.

**Methods:**

Eight judges were recruited and trained. Thirteen 1-hour sessions took place over three months. The steps followed were the selection of commercial reference, generation of descriptors, training of the panel, validation of the trained panel and product characterisation. The judges chose one commercial reference (using simple hedonic evaluation) to serve as a reference. The accepted intensity scale for the generated descriptors was from 0 (low intensity) to 9 (very intense). To test the first versions of the game, food product developers involved in the project, acted as participants, and used a mix of lentils, quinoa, and oats to enhance the commercial version. R-project software was used to analyse the performance of the panel and the sensory profiles.

**Results:**

A glossary with 14 descriptors was generated. The discriminatory capacity of the panel was confirmed by examining the significance of the product effect (p < 0.05). The product–judge interaction was not significant (p > 0.05) for most of the evaluated attributes, indicating a good degree of panel agreement. Overall, the panel was considered reproducible after 9 sessions. Although the appearance, firmness, fragility and chewiness were considered similar to the reference, juiciness and taste (understood as meaty flavour) of the new product were deemed improved.

**Conclusions:**

According to the panel, two of the most appreciated attributes associated with meat analogues, juiciness and taste, were improved compared to the commercial reference. Therefore, the first approach for further development of the contest/game was validated.

## Plain language summary

Don't you think it's important for a child to experiment with food from a very young age? Do they know where the food comes from? How do the products arrive to the supermarket? If the answer is yes and if your kids believe that meatballs can only be made with meat, this game is for them, and for you. "Imagine a future generation of children who, from a young age, have learned that vegetables are not just an alternative to meat, but a wholesome choice for nutrition and health".

This is study is part of the TITAN project, where different gaming options using artificial intelligence will be tested to promote healthy nutritional habits in scholars aged 6–12 years. One of these games is being designed as a contest were students have to develop a ready-to-eat meal, similar to “meatballs in sauce” but using only plant-based ingredients. They will have to improve the sensory properties of a commercial reference. But, to establish a common criterion for assessing the results, it is necessary to develop a common lexicon with descriptors that a trained panel can use to assess the improved prototypes. In this study, 8 judges (4 men and 4 women) were recruited and trained to develop a sensory profile for this type of products. To test the first versions of the game, food product developers involved in the project, acted as participants, and used a mix of lentils, quinoa, and oats to enhance a commercial version. The panel of trained experts validated the results.

## Introduction

Healthy nutritional habits start by educating children and their families. Providing educational content of interest to children and their parents using gaming technologies is a promising tool that needs further development. For example, competitive-cooperative games where improving the nutritional profile a particular meal is the challenge to be solved, can influence peer pressure ^1^. During the TITAN project, a particular challenge related to improving the nutritional and sensory profile of ready-to-eat meals is being tested before developing a gaming tool for kids and parents. The challenge will be presented as follows.

The large-scale production of animal proteins is considered a strong driver of the loss of biodiversity, global climate change and shortage of water ^2^. With the increasing world population and growing concern for animal welfare and environmental properties, the demand for the transition from an animal-based to a plant-based diet is rising.

One of the many possible solutions to the current and future health and environmental challenges is to lower meat consumption and promote plant-based foods ^3^. Substituting animal products with alternative proteins could be a viable strategy. However, introducing plant based meat alternatives faces many difficulties despite the known health and environmental issues associated with meat production ^4^. The aversion of consumers to these dietary changes, often associated with the sensory and nutritional appeal of meat-based foods and simple access to such products, are of prime concern ^5, 6^.

The plant-based meat analogues have attracted much attention thanks to their established health claims and functionality. Moreover, the required nutritional value can be achieved by modifying the physical properties of these products and, at the same time, promoting their sustainability over sensory descriptors ^7^.

Many companies have begun to explore the replacement of animal meat-based ingredients with alternative proteins. Some plant-based products are already accessible on the market, such as chunks, strips, patties, sausages, chicken-like blocks, nuggets, ground beef-like products, meatballs and steaks ^8^.

The biggest challenge facing the developers of meat analogues is obtaining meat-like texture and flavour ^9^. The difficulties in recreating these sensory properties using plant protein sources are usually caused by strong flavours of legumes and decreased tenderness and succulence caused by reduced saturated fat content. However, protein blends can create synergies to overcome these technical issues ^10, 11^.

Soy protein has been the most common base ingredient, but the use of other ingredients like pea and proteins from a range of grains and legumes are expected to increase ^12^. Protein sources such as quinoa, lentils and oats have been reported as sustainable protein sources ^13^ with potential beneficial effects for human health ^14^. However, even though most of them are already on the market, they are rarely used in everyday foods.

The psychochemical, functional and sensory characteristics of meat analogues can be manipulated to resemble those of meat-based products by changing their texture, flavour, appearance, mouthfeel, digestibility and bioavailability of the nutrients ^15^. Testing the sensory properties of such novel products and their optimisation based on customer driven evaluation plays a decisive role in their development and commercialisation ^16^.

Several studies have reviewed consumer acceptance of alternative proteins, innovative food and novel food technologies ^17^ and meat consumption ^18^. A review of consumer research on meat replacements and specific alternatives, such as cultured meat and insect and plant-based substitutes, is also available ^19^.

Recently, many alternative protein sources (e.g., plants, insects or fungi) have been explored as replacements for animal‐derived proteins ^20, 21^. However, a lack of familiarity with novel foods affects expectations and can negatively impact sensory perception and overall liking ^22^. It is important to remember that the opinion of the consumer can also be affected by personal factors, not just the sensory properties of the product.

The texture of meat has been widely studied. Many mechanical methods, analytical techniques and sensory evaluations are established for meat and fish products; however, it is unclear whether these methods are adequate for characterising the plant-based matrices ^23^. Very little information is available on the flavour profiles of products with high content of plant proteins in comparison with meat such as beef, chicken or pork. Almost all available analytical methods deal with technical aspects and are not directly linked to sensory descriptors. In contrast, the sensory methods can provide both quantitative and qualitative data on flavour, taste, texture and appearance ^8^.

The techniques used in sensory evaluations offer data beyond the oral perceptions of food. It is important to note which characteristics contribute to the acceptance of the product itself and which differences are associated with personal factors. Descriptive analysis is the most accurate method; it delivers both qualitative and quantitative data on the sensory profiles supplied by a trained panel. Such panels can provide the description of sensory characteristics and differences between the products, the qualification of intensities and recognition of descriptors ^8^. A panel is considered expert when all the panellists can determine differences, reproduce the results, and are consistent with the rest of the panel. This technique improves the efficiency of the development of analogous meat products.

The plant-based meat analogues have some common flaws, e.g., lack of tenderness and characteristic juicy mouthfeel of meat. Some changes in processing technology and formulation of meat analogues are essential to overcome these shortcomings. There are some reports on the formulation of hybrid ^24^ or fully plant-based meatballs ^25– 27^, but the processes described do not include sterilisation to obtain a shelf-stable precooked meal. The methods used in these studies use a cooking process on a laboratory scale. The current study employs processing methods on a pilot scale.

Here, the lexicon appropriate for the analysis of the studied products was developed, and an expert panel trained to the required standards. This panel conducted the validation and sensory profiling of newly formulated product (ready-to-eat plant-based meatballs in tomato sauce, containing a blend of lentils, quinoa and oats).

## Methods

### Preparation of the ready-to-eat plant-based meatballs in tomato sauce

Ingredients: The formulation of the plant-based meatballs was carefully designed based on (i) their nutritional compositions, contributing to the overall balance of macronutrients (protein, carbohydrates, and fat) as well as the presence of key micronutrients in the plant-based meatball formulation, and (ii) to mimic the sensory properties of meat while using only plant-based ingredients. The primary ingredients chosen were a blend of red lentil flour, quinoa, and oat flour. Red lentil flour (25% protein, source of dietary fiber, folate, iron, potassium and zinc) was selected for their high protein content and texture that closely resembles meat when cooked. Quinoa (15% protein, source of, dietary fiber, iron, magnesium, and phosphorus) was included for its nutritional value, particularly its high protein content and essential amino acids profile. Oat flour (10% protein, source of dietary fiber, beta-glucan, magnesium, phosphorus, and antioxidants) was used for their ability to improve the texture and to bind the ingredients together, contributing to the meatball's firmness and chewiness.

The proportions of these ingredients were determined through a series of preliminary sensory evaluations and trials. The aim was to create a product that not only resembled meat in terms of texture and flavour but also improved upon certain sensory attributes such as juiciness and taste. The final formulation consisted of water (17.0%), textured soy protein (17.0%), quinoa (5.0%), red lentil flour (5.0%), oat flour (1.0%), breadcrumbs (2.0%), soy sauce (1.0%), olive oil (1.5%), garlic powder (0.2%), salt (0.1%), parsley (0.1%), black pepper (0.1%) and tomato sauce (50%; consisting of water, tomatoes, sunflower oil, onion, sugar, salt and citric acid). All the ingredients were bought in a local supermarket (Makro, Erandio, Spain) except for the red lentil flour made at AZTI’s pilot plant (Derio, Spain) using the lentils bought at the same supermarket. An ultracentrifuge mill (ZM 100, Retsch, Haan, Germany) with a sieve of 500 μm was used for producing the flour.

Production process: The ingredients were weighed and mixed in the Kenwood food processor at minimum speed. Once mixed, the mass was manually formed into balls. The balls weighed between 9 and 11 g. Afterwards, 6 balls were packed in aluminium vacuum bags (160 × 270 × 52 mm; Bolsaplast, Barcelona, Spain) and covered with tomato sauce. The bags were sealed under a vacuum of 750 mbar (Multivac C 200, Multivac, Wolfertschwenden, Germany). 

The sterilisation process was performed in a pilot retort (Model APR-95, Surdry, Vizcaya, Spain) programmed to achieve 110°C in 15 min, hold this temperature for 60 min and cool to 30°C in 13 min. The pressure was programmed to the maximum of 0.8 bar. The temperatures were recorded employing a TrackSense Pro Mini Wireless Data Logger Serial No. 84633 (Ellab, Hillerød, Denmark) using the ELLAB software ValSuite Basic 3.1.3.10v (Ellab, Hillerød, Denmark). Data acquisition was performed at intervals of 1 min. In each batch, 1 "meatball" was punctured with the data logger before sealing the bag. The temperature at the core of the product was maintained at 105—108°C for 34 min, achieving an F _0_ of 3. Then, the samples were stored at ambient temperature.

### Sensory analysis

Sensory tests were carried out in a standard tasting room ^28^ with ten individual taste booths separated by screens to isolate the different judges. Samples were served at room temperature (about 20°C) on white plates. The sensory rooms were equipped with full-spectrum daylight LED lighting, which ensures consistent illumination across evaluations.

The ethical approval was conducted according to quality standards of the ISO 8586:2012 certified by AENOR, the Spanish Association for Standardization and Certification.

### Recruitment of judges and basic training

The recruitment process was carried out to attract and train sensory assessors who were also consumers of meat analogues. Non-consumers were excluded to focus on feedback from potential regular consumers of plant-based products. An email was sent to all AZTI employees (> 290) explaining the objective of the study and the profile required (female or male consumers of plant-based meat analogues). Those who expressed interest in joining the study were informed of the methods and the time needed to participate. Screening tests to evaluate the sensory abilities of the participants were carried out (a basic taste test, a smell recognition test and a scale management test) ^29^. No remuneration was offered. Participants signed an informed consent prior to participating in the study.

### Demographic characteristics

Eight participants were enlisted, 4 men and 4 women, aged from 27 to 40, all with middle income, employed, and consumers of this type of product at least once a week. Gender balance was aimed to gather a wide range of sensory feedback, reflecting a varied trained sensory assessor base. Eight assessors were chosen based on the availability and resources, acknowledging the deviation from ISO 8586:2023's recommendation of a minimum of 12. 

### Generation of descriptors and training of judges

The specific judge training was performed according to international standards ^25^. Thirteen 1 hour sessions took place over three months. The subjects covered were the selection of commercial reference (1 session), generation of descriptors (2 sessions), training of the panel (7 sessions), validation of the trained panel and product characterisation (3 sessions).

**Figure 1.**
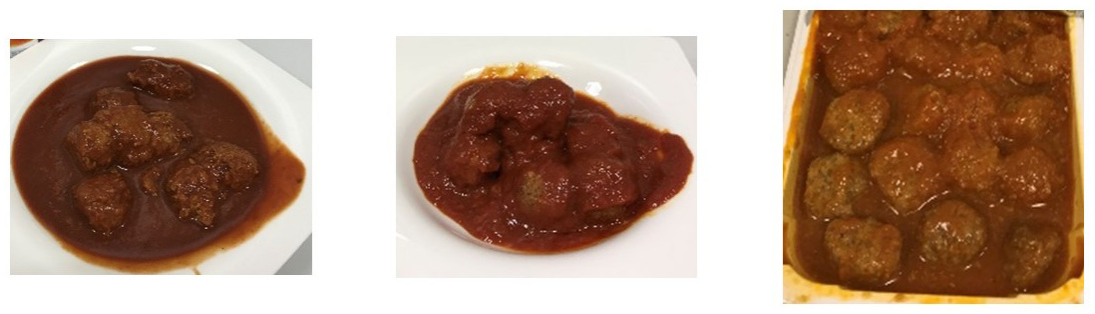
Images of the three commercial plant-based "meatballs" used in the focus group.

A combination of methodologies was used for generating the descriptors. The first qualitative study (face-to-face focus group) was conducted to choose the reference product. The trained sensory assessors were presented with three commercially available types of ready-to-eat plant-based meatballs in tomato sauce ( Figure 1). The focus was on developing a distinct plant-based product rather than directly imitating meat-based meatballs. The selection of these reference products was based on a thorough market search conducted in five local supermarkets (Bilbao, Spain) and three online platforms where these products were readily available for shopping in Spain. This comprehensive approach allowed us to capture a diverse range of shelf-stable plant-based meatballs in tomato sauce options, present in both physical and online retail spaces. The participants tasted the products and supplied a simple hedonic evaluation ("which one do you like most?" and "what is the sample most similar to its analogue?"). On the basis of the results, the trained sensory assessors decided which product would serve as the reference in the subsequent sessions. A standardised procedure for sensory analysis was used, following ISO 11035:1994 ^30^.

The ingredients for each product are listed below.

PRODUCT 1:

Sauces (tomato, onion, carrot, extra virgin olive oil, cane sugar, garlic (citric acid), salt); artificial meatballs (soybean pulp, soybean curd (water, soybeans, magnesium chloride)); sunflower oil, soy, wheat flour, brown rice, cassava starch, oat flakes, parsley, salt, garlic; vegetable broth (onion, carrot, leek, garlic, celery, rice flour, salt, sunflower oil, extracts of yeast, onion powder, carob).

PRODUCT 2:

Filtered water, textured soy protein, isolated soy protein, hydrolysed soy protein, wheat gluten, canola seed oil, tomato juice, modified starch, salt, onion, garlic, vegetable seasonings, and spices.

PRODUCT 3:

Sauce (71%): tomato (40%), water, onion, green pepper, extra virgin olive oil, panela, garlic, pink Himalayan salt and tapioca starch. Meatballs (29%): natural tofu (water, soybeans and magnesium chloride (nigari)), quinoa (20%), textured soy protein, gelling agents: agar–agar and carrageenan, soy sauce (water, soya in variable proportions, 33–46%, sea salt and koji), yeast and Himalayan pink salt.

**Figure 2.**
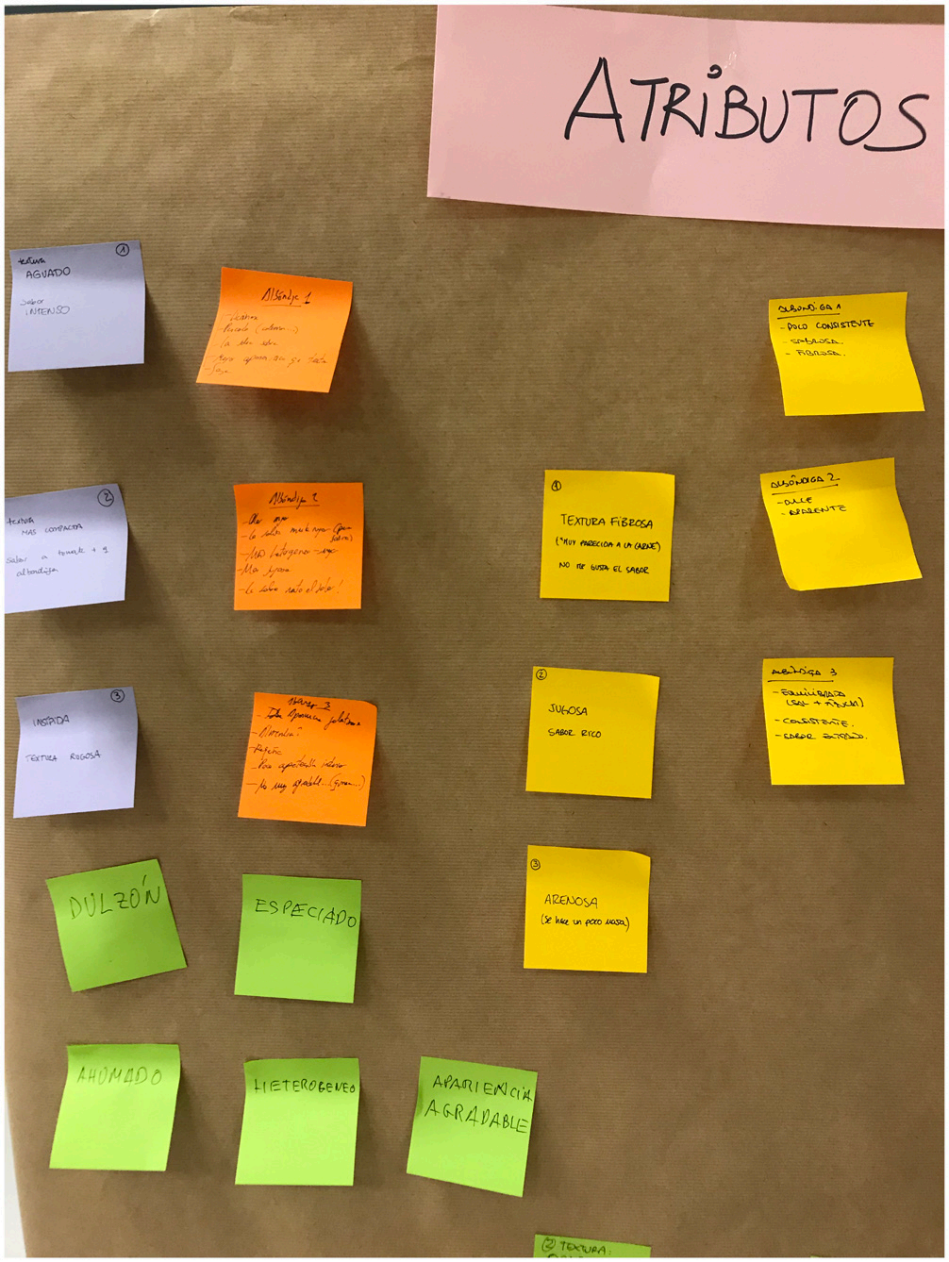
Generation of descriptors for the product selected as the reference.

The methodology for reaching consensus on the final organoleptic attributes in the study involved a structured and participative approach. We conducted a series of training sessions (S1-S6) with the trained sensory assessor to develop a comprehensive sensory lexicon for the selected commercial product, which served as the reference ( Figure 2). To facilitate the process, each trained sensory assessor individually wrote down the descriptors on post-it notes, capturing their sensory perceptions. These descriptors were then collectively pasted onto a common panel, allowing for group discussion and consensus-building. This collaborative approach encouraged active participation and ensured that a comprehensive set of descriptors was generated to capture the sensory attributes of the reference product accurately.

The subsequent training sessions (S3, S4, S5, and S6) built upon this foundation to refine these descriptors further, utilising the agreed-upon descriptors to evaluate the sensory characteristics of the plant-based meatballs.

A structured scale of nine points 24 was used; the descriptors were awarded scores between 1 ("I dislike it extremely") and 9 ("I like it very much"). Each judge was given 2 identical meatballs without tomato sauce to focus on the meatball taste. In the sensory analysis of plant-based meatballs, a critical step involved the removal of the sauce to focus solely on the meatball's inherent sensory properties. This was achieved through a careful and standardized process using a napkin. Each meatball, initially prepared with sauce, was gently lifted from its sauce. Then, using a napkin, the surface of the meatball was dabbed to absorb the sauce. This dabbing was performed with enough care to ensure that the sauce was absorbed without exerting pressure that might alter the texture of the meatball. The goal was to remove any visible traces of the sauce, effectively isolating the meatball from external flavor influences. This method ensured that each meatball was treated uniformly, allowing for an accurate and focused assessment of its intrinsic sensory characteristics. Through iterative sessions and discussions, the trained sensory assessor achieved a consensus on the intensity of each descriptor for the reference product after seven sessions. The final organoleptic attributes were standardized through a consensus-based approach among the participants.

This rigorous training process ensured the panel’s ability to evaluate and discriminate the sensory characteristics of the plant-based meatballs accurately.

### Performance of the panel and sensory profile development

The panel validation was based on ISO 11132:2021 (Guidelines for the measurement of the performance of a quantitative descriptive sensory panel) ^31^. For each descriptor, the consistency of the panel between sessions was measured. After each tasting session, all responses were pooled to identify the descriptors and judges whose standard deviation was ≥ 1. The difference between sessions should not be significant at the 0.05 level; otherwise, it would mean that the scores of the individual tasters were inconsistent. The participants also scrutinised the descriptors that might be irrelevant or difficult to measure and discussed the potential harmonisation of sensory evaluation for the product. After 7 sessions, the consensus on the intensity of each descriptor of the benchmark commercial product was reached (agreed QDA).

Once the panel performance was deemed repeatable and reproducible, and an adequate capacity of discrimination was determined, the sensory evaluation of the ready-to-eat plant-based meatballs in tomato sauce was conducted. Each assessor was provided with the 2 samples, the reference and the improved prototype (2 pre-heated meatballs per product without tomato sauce on a plate), coded with a 3-digit random number. Sample evaluations were performed at room temperature under normal lighting conditions. All samples were assessed in random order in three sessions (P1, P2 and P3). The trained sensory assessors scored the intensities of the generated descriptors. The results were analysed to determine whether there were significant differences between the samples and which descriptors made them different. The results were also examined for any resemblance to the QDA of the commercial product.

### Statistical analyses

The data analysis was carried out using R-project software (v 4.1.2). The packages used were "readxl", "rapportools", "tidyverse", "ggplot" and "spiderchart". A one-way ANOVA was conducted (per trained sensory assessor) at the end of each session to see if there were any statistically significant differences (p < 0.05) between the assessors, and a three-way ANOVA to analyse statistical significance (p < 0.05) of the trained sensory assessor, descriptor and session.

## Results and discussion

### Glossary of descriptors for plant-based meatballs

Results were obtained and examined in 4 steps. First, the focus group results showed that the most preferred sample was "product 3", voted as the favourite by all trained sensory assessors. In the second step, two sessions of open discussion (S1 and S2) were organised to generate the list of descriptors and the procedure for evaluating their intensity ( Table 1).

**Table 1.** Descriptors and definitions used to evaluate the commercial ready-to-eat plant-based "meatballs" in tomato sauce.

Descriptors	Definition and procedure used for their evaluation
**Visual**	
Outer colour	1, raw meat colour; 5, medium meat colour; 9, light brown
Brightness	1, low brightness; 9, high brightness
Elasticity	The middle of the meatball will be pressed with a thumb, slightly indenting the sample (pressing down to between 1/4 and 1/5 of the thickness). The recovery of the initial shape of the meatball will be assessed. The sample is considered very elastic and will be assigned a high score if it recovers quickly.
Inner colour	1, raw meat colour; 5, medium meat colour; 9, light brown
Compactness	The internal appearance of the meat: 1, loose-consistency meat and 9, very compact meat.
**Odour**	
Characteristic odour	The higher the intensity, the higher the score assigned (within the scale).
**Texture**	
Firmness	Force required to break the sample with the first bite. Bite the meatball in half with your incisors and assess the force exerted; 1, little force (like biting through a croquette) and 9, much force needed (for example, like biting through an almost raw chop).
Cohesiveness/Fragility	The ease with which the sample can be broken: 1, a very hard meatball, difficult to break; 9, a meatball that breaks easily without applying much force.
Juiciness	The sensation of moisture in the mouth during chewing. The greater the sensation of juiciness, the higher the score.
Chewiness	The number of chews necessary to swallow the meatball (1, very chewy, e.g., like black pudding; 9, not very chewy, e.g., tough meat).
Adherence	Force required to detach the product from the palate (1, not very adherent; 9, very adherent).
Graininess	Detection of granules or small particles
Pastiness	The effort required to swallow the product (1, a little; 9, very doughy)
Fat perception	The sensation of fatty coating in the mouth, warm sensation: 1, weak fat perception (e.g., chicken breast); 9, strong fat perception (e.g., bacon).
**Taste**	
Characteristic taste	Meaty flavour
Aftertaste	The higher the persistence, the higher the score assigned (within the scale).

**Figure 3.**
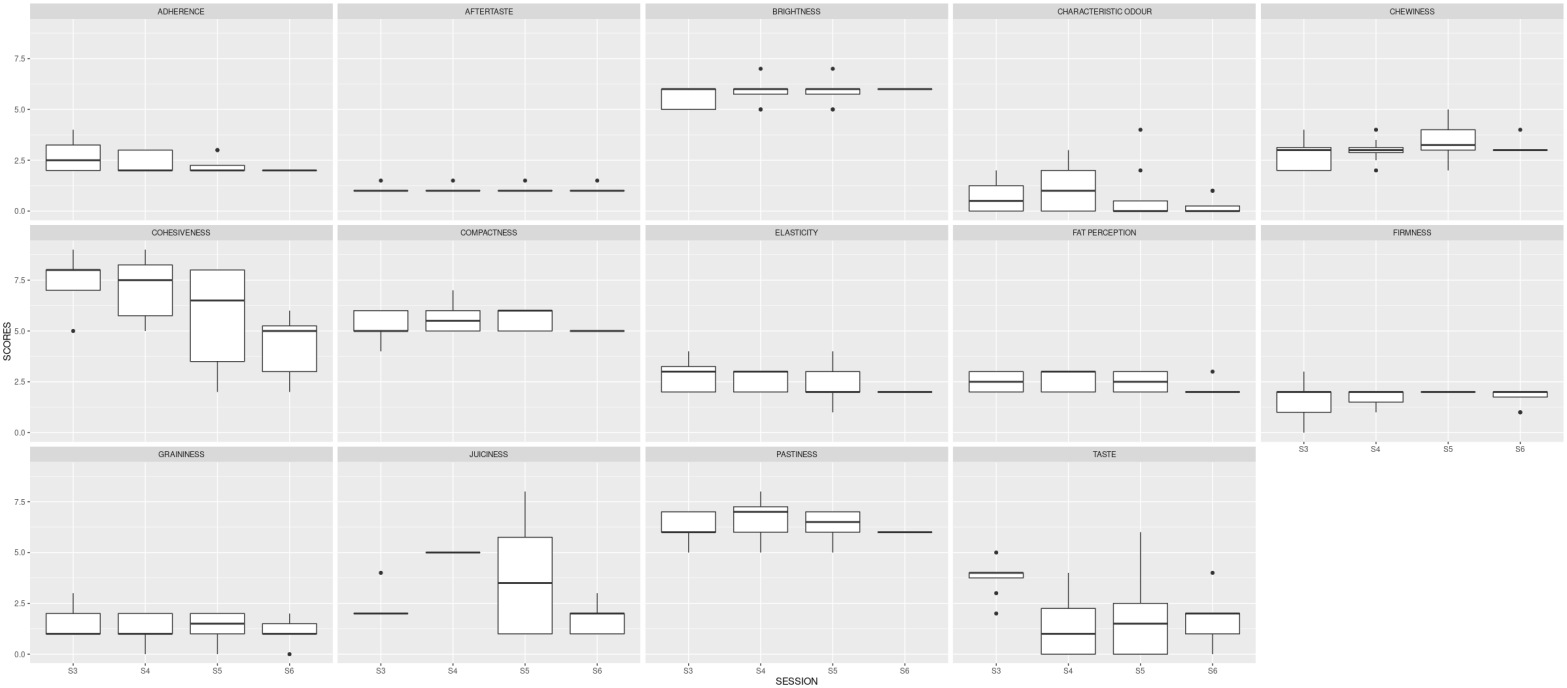
Boxplots showing the sensory results per judge and descriptor in Step1 (4 sessions).

In the second stage, the panel was trained to assess the intensity of each descriptor during four sessions (S3, S4, S5 and S6). The product tested was the commercial reference. The results of these first four sessions are shown in Table 2 and Figure 3. The ANOVA carried out to examine descriptor scores assigned in different sessions showed very significant differences (p < 0.01) for cohesiveness and juiciness and significant differences (p < 0.05) for taste. In fact, the standard deviation for these descriptors per trained sensory assessor was > 1. To confirm the repeatability of a trained sensory assessor in different sessions for the same descriptor, the standard deviation should be < 1.

**Table 2.** Sensory results per judge and descriptor in Step 1 (4 sessions).

Descriptor	Coefficients							Statistics		
	Judge 1	Judge 2	Judge 3	Judge 4	Judge 5	Judge 6	Judge 7	Judge 8	p-value	Mean panel ± SD
Brightness	5.75±0.50	5.75±0.50	6.50±0.58	6.00±0.00	5.50±0.58	5.75±0.50	5.75±0.50	5.75±0.50	0.545	5.84±0.51
Elasticity	1.75±0.50	2.25±0.50	3.00±0.82	2.25±0.50	3.00±0.82	2.50±0.58	2.50±0.58	2.50±1.00	0.082	2.47±0.72
Compactness	5.00±0.82	5.00±0.00	5.25±0.50	5.50±0.58	6.00±0.82	5.25±0.50	5.25±0.50	5.75±0.50	0.102	5.38±0.61
Characteristic odour	0.25±0.50	0.00±0.00	0.50±1.00	0.50±1.00	0.50±1.00	1.50±0.58	2.50±1.29	0.00±0.00	0.4707	0.72±1.08
Firmness	2.00±0.00	1.50±0.58	1.63±0.48	2.00±0.00	2.00±0.82	1.75±0.50	2.00±0.00	1.38±0.95	0.582	1.78±0.54
Cohesiveness	6.00±2.94	5.75±1.50	7.25±1.50	4.00±2.45	7.00±1.41	6.00±2.45	5.75±2.87	7.50±1.29	0.008 **	6.16±2.17
Juiciness	2.50±1.73	3.25±1.71	2.75±1.50	2.25±1.89	4.25±2.87	4.25±2.87	3.25±2.06	3.25±2.06	0.001 **	3.22±2.01
Chewiness	3.00±1.41	3.00±0.00	2.88±0.63	3.13±0.48	3.00±0.00	4.00±0.00	3.00±0.82	2.75±0.50	0.334	3.09±0.69
Adherence	2.25±0.50	2.75±0.96	2.00±0.00	2.50±0.58	2.75±0.50	2.50±1.00	2.00±0.00	2.00±0.00	0.083	2.34±0.60
Graininess	1.25±0.50	0.25±0.50	1.50±0.58	1.25±0.50	1.75±0.50	1.00±0.00	1.67±0.58	2.00±0.82	0.795	1.32±0.70
Pastiness	5.75±0.50	5.50±0.58	6.25±0.50	7.00±0.82	6.50±0.58	6.50±0.58	6.25±0.50	7.00±0.82	0.244	6.34±0.75
Fat perception	2.50±0.58	2.00±0.00	2.63±0.48	2.25±0.50	2.13±0.25	3.00±0.00	2.50±0.58	2.50±0.58	0.1939	2.44±0.49
Taste	2.50±1.29	3.25±2.75	1.50±1.91	2.25±1.26	0.75±1.50	2.25±1.26	3.50±1.00	1.50±1.91	0.020 *	2.19±1.73
Aftertaste	1.75±0.50	1.50±0.58	1.00±0.82	0.75±0.50	1.00±0.00	0.75±0.50	1.00±0.00	0.75±0.50	1	1.06±0.56

Significance codes for p-value: **, (0.001 < p-value < 0.01); *, (0.01 < p-value < 0.05), (0.05 < p-value < 1).

Three more sessions were needed to reduce the variability between the judges. Table 3 shows the results of the two-way ANOVA test for each judge and session; after the three additional sessions, the panel was consistent. The variability of each judge was reduced, and the standard deviation per descriptor and per judge was < 1.

**Table 3.** Evolution of descriptors defined during the qualitative descriptive analysis of the benchmarked product (7 sessions).

STEP 1	STEP 2	
S3, S4, S5, S6	S7, S8, S9	AGREED QDA
Brightness	- Brightness	+Inner colour
		+Outer colour
Elasticity	Elasticity	Elasticity
Compactness	- Compactness	
Odour	Odour	Odour
Firmness	Firmness	Firmness
Cohesiveness	Cohesiveness	+Fragility
Juiciness	Juiciness	Juiciness
Chewability	Chewability	Chewability
Adherence	Adherence	Adherence
Graininess	Graininess	Graininess
Pastiness	Pastiness	Pastiness
Fat perception	Fat perception	Fat perception
Flavour	Flavour	Taste
Aftertaste	Aftertaste	Aftertaste

Figure 2 also depicts the reduced variability, illustrated by the size of the boxplots. The black dots represent the outliers, the solid lines show the median value of the descriptor, and the whiskers represent the minimum and maximum values.

The three descriptors with high variability in the first analysis (cohesiveness, juiciness and taste) were more homogeneously assessed by the 8 judges during the subsequent 3 sessions.

The additional three sessions (S7, S8 and S9) were conducted to re-train the trained sensory assessors for the chosen descriptors. A particular emphasis was placed on the definition and the intensity of these descriptors. Table 3 shows the changes in the descriptors generated in Step 1 (retained, renamed or deleted).

The panel had difficulties evaluating brightness because the tomato sauce was removed from the meatballs during the tasting session (to reduce the effect of the sauce on the taste). The participants found it easier to assess the external and internal colour of the product (the latter was not affected by the sauce).

Another attribute that was difficult to assess was compactness as the panel did not understand the difference between compactness and cohesiveness or firmness. Therefore, the term "firmness" was retained, and "compactness" was removed. Following the same trend, cohesiveness was removed to avoid confusion, and "fragility" was included as the opposite of compactness/cohesiveness.

Finally, the flavour was deemed an overly complex attribute to assess; it was agreed to replace it with taste (understood as a meaty flavour in English).

In English, the terms "taste" and "flavor" have distinct meanings, which can lead to confusion in translation, especially into Spanish.

Taste: This refers to the sensation produced when a substance in the mouth reacts chemically with taste receptor cells located on taste buds. Taste is limited to five basic qualities: sweet, sour, salty, bitter, and umami. These basic tastes are perceived through the tongue.

Flavour: It is a broader concept that includes not only the basic tastes but also the aromas and other sensations (like spiciness or temperature) perceived through the olfactory receptors in the nose. Flavour is the overall perception of a food or drink, combining taste, smell, and other sensory attributes.

When translating these terms into Spanish, the distinction can become blurred:

"Taste" is translated to “gusto” or “sabor.” “Gusto” aligns more with the English "taste," referring to the basic taste sensations. However, “sabor” can be interpreted as both “taste” and “flavour,” encompassing the broader sensory experience.

"Flavour" is often translated as “sabor,” but this doesn't fully capture the complexity of the term as understood in English, which includes aroma and other sensory experiences beyond just taste.

The confusion arises because “sabor” in Spanish can mean both the basic tastes perceived by the tongue and the overall flavour experience, which includes aroma and other factors. This overlap in meaning can make it challenging to convey the distinct concepts of "taste" and "flavour" from English into Spanish accurately.

In the context of this study conducted in Spain, the term "taste" (or "gusto" in Spanish) can often be understood to encompass what is referred to as "overall flavour" in English. This broader interpretation in the Spanish context arises from linguistic and cultural nuances in how sensory experiences are described. Therefore, in this study, when the term "taste" is used, especially given its Spanish context, it is aligned more with the English concept of "overall flavour." This encompasses a more holistic sensory assessment rather than just the basic taste sensations. Such linguistic and cultural differences are important to consider in sensory studies, as they can influence how sensory data is described, interpreted, and communicated.

**Figure 4.**
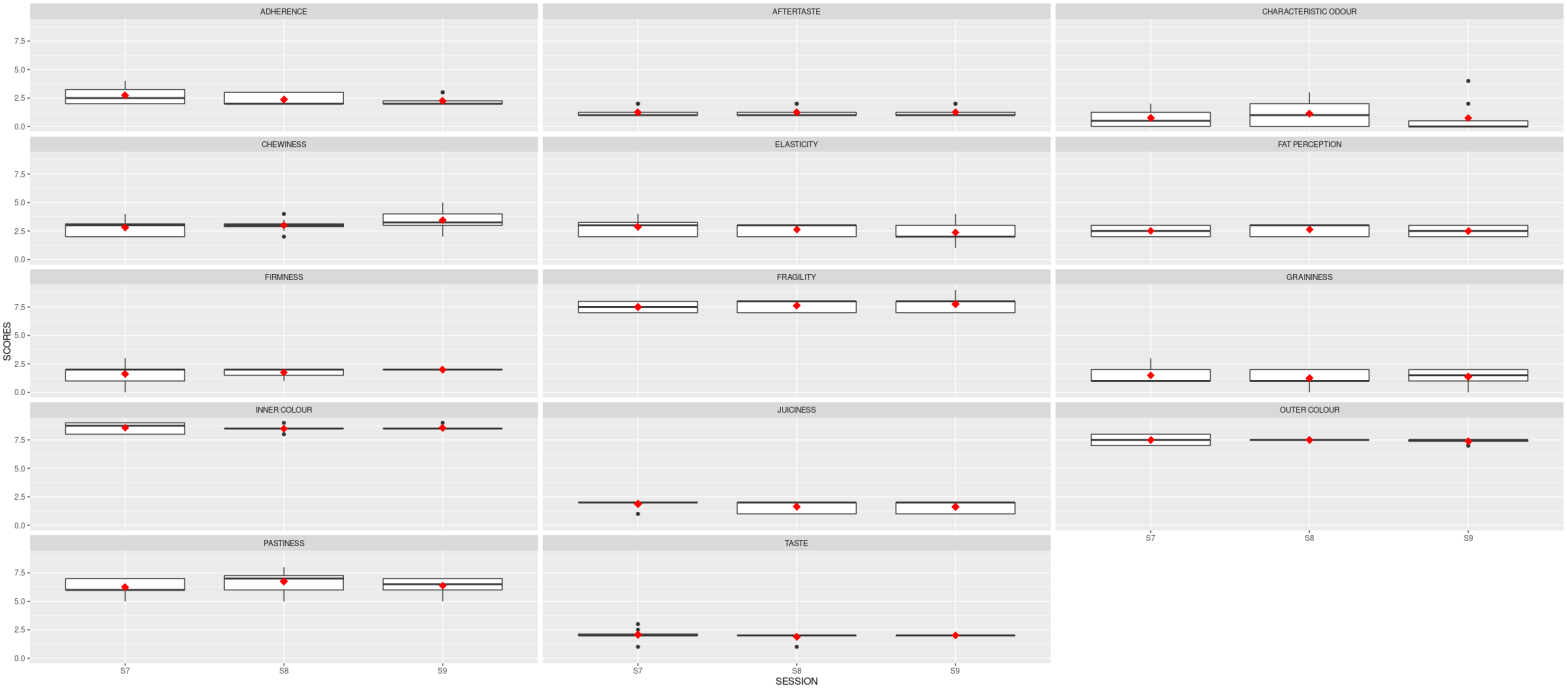
Boxplots showing the sensory results per judge and descriptor in Stage 2 (3 sessions). S7, S8 and S9 are the session names. Red squares show the averages; black dots represent the outliers.

A second ANOVA analysis was carried out to examine the statistical differences and the robustness of the panel after 9 sessions. The results are shown in Table 4. Cohesiveness, juiciness and taste have reduced their variability after the second training. All the standard deviations were below 1. Figure 4 depicts the boxplots for each descriptor per session. The red squares indicate the averages for the panel, and the black dots show the outliers. Noticeably, the red symbols are now aligned, with slight variability per session.

**Table 4.** Sensory results per judge and descriptor in Step 2 (3 sessions).

Descriptors	Coefficients							Statistics		
	Judge 1	Judge 2	Judge 3	Judge 4	Judge 5	Judge 6	Judge 7	Judge 8	p-value	Mean for the panel ± SD
Outer colour	7.31±0.32	7.71±0.35	7.32±0.29	7.33±0.34	7.52±0.02	7.74±0.35	7.73±0.34	7.23±0.35	0.633	7.46±0.21
Inner colour	8.34±0.28	8.74±0.33	8.34±0.34	8.70±0.30	8.73±0.34	8.22±0.28	8.80±0.32	7.24±0.34	0.914	8.36±0.53
Elasticity	1.67±0.58	2.33±0.58	3.33±0.58	2.33±0.58	3.33±0.58	2.67±0.58	2.67±0.58	2.67±1.15	0.449	2.63±0.77
Characteristic odour	0.33±0.58	0.00±0.00	0.67±1.15	0.67±1.15	0.67±1.15	1.67±0.58	3.00±1.00	0.00±0.00	0.783	0.88±1.19
Firmness	2.00±0.00	1.33±0.58	1.50±0.50	2.00±0.00	2.33±0.58	2.00±0.00	2.00±0.00	1.17±1.04	0.425	1.79±0.57
Fragility	7.67±0.58	7.33±0.58	8.00±0.00	7.33±0.58	7.33±0.58	7.67±0.58	8.00±1.00	7.67±0.58	0.705	7.63±0.58
Juiciness	1.67±0.58	1.67±0.58	2.00±0.00	1.67±0.58	1.00±0.00	1.67±0.58	2.00±0.00	2.00±0.00	0.482	1.71±0.46
Chewiness	3.00±1.73	3.00±0.00	2.83±0.76	3.17±0.58	3.00±0.00	4.00±0.00	3.00±1.00	2.67±0.58	0.264	3.08±0.78
Taste	2.00±0.00	2.17±0.29	2.00±0.00	2.00±0.00	2.00±0.00	2.00±0.00	2.33±0.58	1.33±0.58	0.616	1.98±0.38
Aftertaste	2.00±0.00	1.67±0.58	1.33±0.58	1.00±0.00	1.00±0.00	1.00±0.00	1.00±0.00	1.00±0.00	1.000	1.25±0.44
Adherence	2.33±0.58	3.00±1.00	2.00±0.00	2.67±0.58	3.00±0.00	2.67±1.15	2.00±0.00	2.00±0.00	0.298	2.46±0.66
Graininess	1.33±0.58	0.33±0.58	1.67±0.58	1.33±0.58	1.67±0.58	1.00±0.00	1.67±0.58	2.00±1.00	0.796	1.38±0.71
Pastiness	5.67±0.58	5.33±0.58	6.33±0.58	7.33±0.58	6.67±0.58	6.67±0.58	6.33±0.58	7.33±0.58	0.478	6.46±0.83
Fat perception	2.67±0.58	2.00±0.00	2.83±0.29	2.33±0.58	2.14±0.29	3.00±0.00	2.67±0.58	2.67±0.58	0.850	2.54±0.49

A review published by Djekic *et al.* ^32^ has reported that the average number of sessions necessary to train a panel can be as high as 10 (in 22.9% of the searched articles) or even higher (in 5% of the cases). However, no data have been provided in 72.1% of the cases. This is a relevant issue as training a panel can take up to 3 months. Reporting the number of sessions or the time necessary for training is essential as it has a bearing on the robustness of the panel and, thus, can affect the conclusions of the sensory analysis.

Once the standard deviations per descriptor and judge were reduced to less than 1, the panel was scrutinised for its robustness, reproducibility, and repeatability ( Table 5).

**Table 5.** F-values in three-way ANOVA (8 judges, 3 products, 3 sessions) for all descriptors.

Descriptors	Product	Judge	Session	Product–Judge	Product–Session	Judge–Session
Outer colour	13.46	3.18	3.00 ***	0.82	0.34	0.32
Inner colour	73.65	0.87	0.90	0.83	1.12	1.60
Elasticity	62.02 **	0.51	0.87	3.26 *	2.18	2.90 *
Characteristic odour	263.28 ***	0.60	1.00	5.02	0.21	1.64
Firmness	3.33	0.21	0.03	2.94	3.28	0.75
Fragility	27.81	1.16	0.36	0.57	1.77	1.34
Juiciness	448.00 *	43.00	0.28	0.26	3.10	0.80
Chewiness	126.44	0.41	2.54	1.07	0.40	1.37
Taste	780.34 ***	0.54	0.36	2.07	2.54	1.44
Aftertaste	3642.03 *	1.83	0.47	1.06	0.54	1.61
Adherence	42.00 *	2.27	1.93	1.42	0.80	1.08
Graininess	1292.85	1.35	26.60	1.41	0.06	0.98
Pastiness	172.50 ***	0.72	0.02	5.75 **	6.24 *	2.15
Fat perception	29.56 *	0.33	0.00	5.71 **	1.00	4.29 **

Significant effects: *p-value < 0.05, ** p-value < 0.01, ***p-value < 0.0001.

## Panel performance

### Discriminatory capacity

A significant product effect means that the judges can discriminate between the products. The p-values ( Table 5, F-values in three-way ANOVA, column 1) indicated that the panel was capable of such discrimination based on most of the descriptors, except for the outer or inner colour, firmness, fragility, chewiness and graininess. Overall, the results confirmed that the panel could distinguish between the products.

The judge effect was not significant (p > 0.05) for any of the descriptors ( Table 5, column 2), which implies that the assessors did not differ in evaluating the products. However, there were significant differences (p < 0.05) between scores given for the outer colour ( Table 5, column 3); i.e., the outer colour varied between the sessions, and it was not repeatable. This might have happened because the procedure for removing tomato sauce differed from session to session.

### Panel agreement

The product–judge interactions ( Table 5, column 5) were not significant (p > 0.05) for most of the evaluated attributes, which indicated a good degree of agreement within the panel. Significant interactions were only observed for 3 descriptors (elasticity, pastiness and fat perception), and overall, good repeatability was achieved. A significant product–judge interaction would imply a lack of consensus for the given variable. Moreover, it is necessary to take into account the product effect; thus, when this effect is also significant, an adequate panel agreement is presumed.

### Panel reproducibility

The product–session and judge–session interactions were analysed to examine the panel reproducibility. Non-significant product–session interactions mean that each product was assessed in the same way in each session. Reproducibility between the different sessions was considered very good for the panel as a whole; significant differences were found only for one descriptor, the pastiness ( Table 2, column 5). This descriptor was scored differently in different sessions. The judge–session interaction ( Table 5, column 6) was only significant for elasticity and fat perception. This implies that some judges did not assign the same scores for those descriptors to all the samples in the two replicates. Overall, the panel reproducibility was very good.

Since the panel response was repeatable and reproducible, and their discrimination capacity was adequate, the judges were considered sufficiently trained, and the sensory evaluation stage could begin.

### Sensory profile of ready-to-eat plant-based "meatballs"

**Figure 5.**
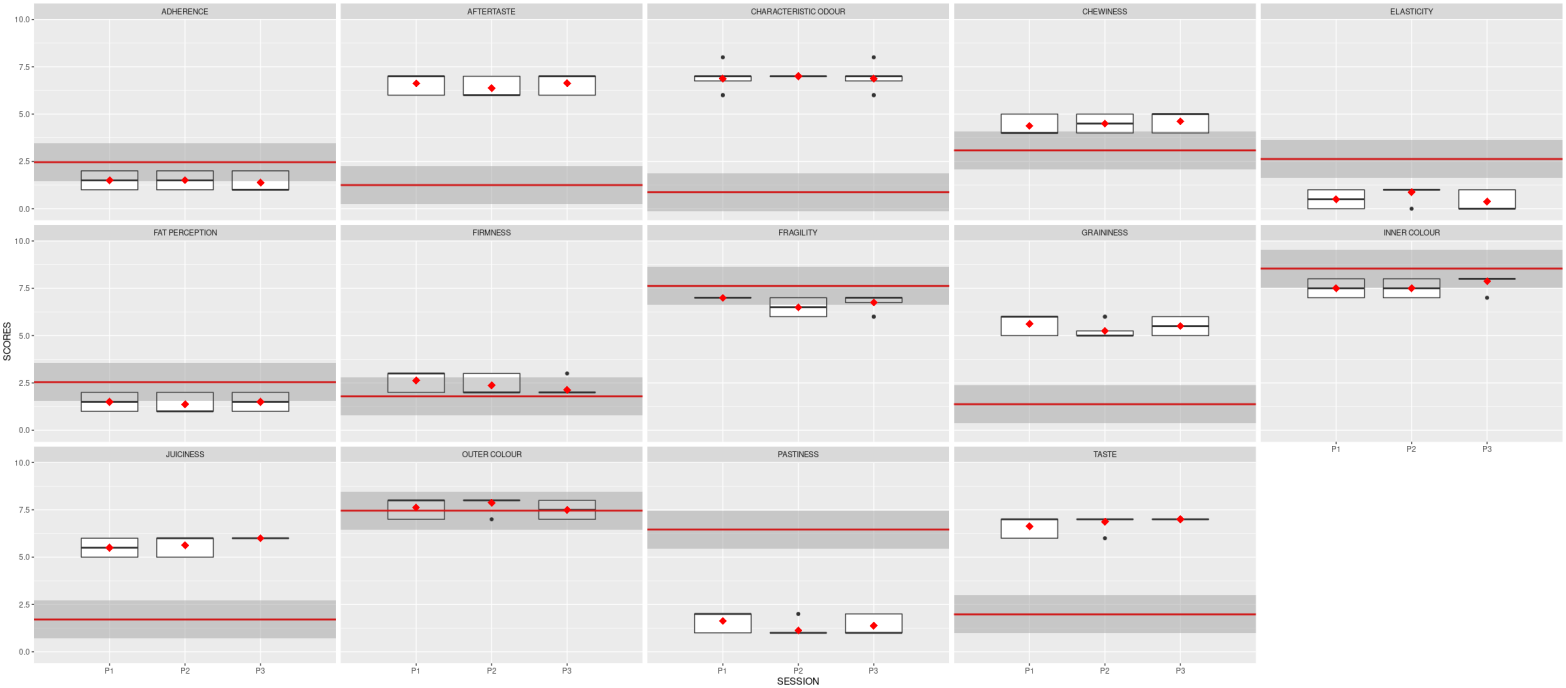
Boxplots showing the sensory results for each judge and descriptor in Step 3 (3 sessions).

The mean results obtained for the two samples analysed are depicted in Figure 5. The red line represents the average value for the reference sample; the dark grey stripe shows an area of ± 1 standard deviation from the agreed QDA for the reference sample. The red squares indicate the mean values for the improved prototype in each session, and the black symbols represent the outliers. Solid lines in the boxes mark the median values of the descriptor, and the whiskers represent the minimum and maximum values. The prototype sample values fell within the reference QDA area for firmness, fragility, inner colour, outer colour, and chewiness.

The ANOVA results for product variability presented in Table 5 (column 1) show significant differences between the prototype and reference samples (p-value < 0.05) for aftertaste (6.5 vs 1.2), adherence (1.4 v 2.4), fat perception (1.4 vs 2.5) and juiciness (5.7 vs 1.7). The values for elasticity (7.6 vs 8.5; p-value < 0.0001), characteristic odour (6.9 vs 0.8), taste (6.8 vs 2.0) and pastiness (1.4 vs 6.4) also differed significantly between these products.

Aftertaste, juiciness, characteristic odour and taste were rated higher for the new prototype samples, i.e., the intensities assigned to these descriptors were stronger for the prototype formulation than for the reference. In contrast, the intensities for adherence, fat perception, elasticity and pastiness were reduced in the prototype; the improved formulation lowered the sensory perception of these attributes.

Appearance-related descriptors (the outer and inner colour) and texture-related descriptors (firmness, fragility, graininess and chewiness) did not differ between the samples.

**Figure 6.**
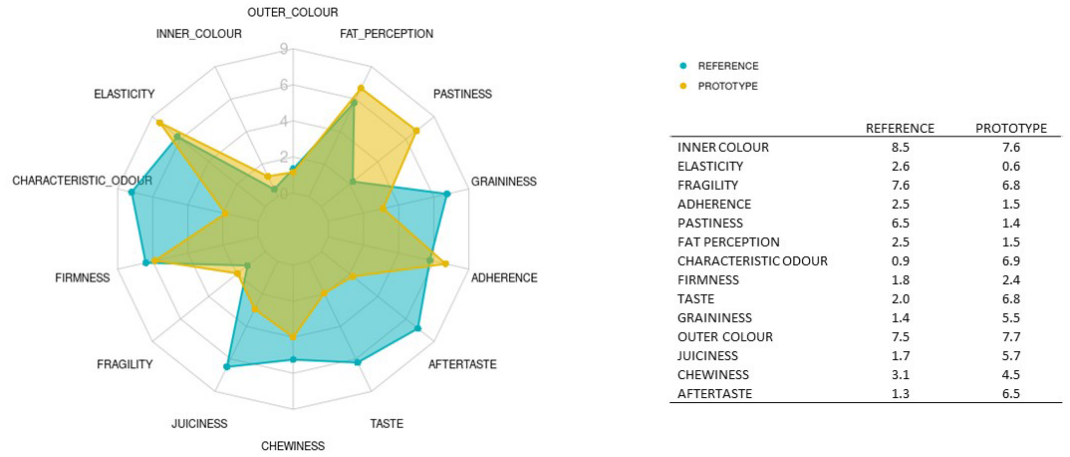
Spider chart showing sensory profiling of the reference meatballs (REFERENCE) and the improved prototype (PROTOTYPE); n = 3 sessions. Mean values are shown in the attached table.

The sensory profiles for the two products are plotted in Figure 6. The statistical significance (p-value) of the difference between the two samples is shown in Table 5. The main difference between the formulations is the use of gelling agents. The commercial reference product contains agar–agar and carrageenan, whereas the new prototype plant-based "meatballs" were made without any gelling agents. This could explain the lower scores assigned to the texture-related descriptors such as adherence, elasticity and pastiness in the prototype samples compared to the reference. The hydrocolloids employed as thickening agents in foods increase the intensity of attributes such as adhesiveness and elasticity ^33^. Carrageenan, used in gel formation, is associated with a perception of pastiness during oral breakdown ^34^. These are some of the undesirable sensory attributes of meat analogues ^35, 36^. The taste-related descriptors of new prototype samples, the aftertaste, juiciness and taste, obtained higher scores than the reference product descriptors. In a study by Godschalk-Broers, Sala ^37^, taste and juiciness were highly correlated with consumer liking. In another recent study by Starowicz, Kubara Poznar ^38^, juiciness and meaty taste have been reported among the main sensory attributes determining the acceptance of meat alternatives.

The fat perception was stronger in the commercial sample than in the new product. Godschalk-Broers, Sala ^37^ also report that large fat globules or pools of oil are associated with stronger fat perception. Although their study focused on chicken-analogue pieces, similar behaviour can be expected when tasting "meatball" analogue pieces elaborated with texturised protein ingredients.

## Conclusion

The study focuses on creating a lexicon to objectively assess the sensory profile of plant-based meatballs. The research involved training eight judges over thirteen 1-hour sessions across three months. The panel was able to develop 14 descriptors for sensory evaluation, with a significant product effect observed for most descriptors, indicating the panel's ability to discriminate between products. The study found that attributes like juiciness and flavour, crucial for meat analogues, were improved in the plant-based product compared to a commercial reference. The sensory profile demonstrated that the developed plant-based product had enhanced attributes like juiciness and taste without compromising other characteristics like appearance and texture. The study emphasizes the critical role of a trained panel in sensory evaluation and presents a robust methodology that can be applied to a broader range of plant-based foods. It lays a foundation for future research aimed at expanding sensory evaluation across diverse food categories and developing specific lexicons for each, thereby contributing significantly to sensory analysis and innovation in the plant-based food sector.

## Data Availability

Zenodo: Fundacion-AZTI/TITAN: Development of the lexicon, trained panel validation and sensory profiling of new ready-to-eat plant-based "meatballs" in tomato sauce. Extended data. http://doi.org/10.5281/zenodo.7452100 ^39^. This project contains the following underlying data: Quali_sessions_panel.xlsx Panel_3steps.xlsx R Sensory panel meatballs.R The qualitative data is in Spanish, however, readers should contact the corresponding author for any queries on the qualitative data. Data are available under the terms of the Creative Commons Attribution 4.0 International license (CC-BY 4.0).
