# Supplementary material for: Development of the lexicon, trained panel validation and sensory profiling of new ready-to-eat plant-based " meatballs" in tomato sauce
Source: Open Res Eur. 2024 Feb 13;2:145. Originally published 2022 Dec 21. [Version 3] doi: 10.12688/openreseurope.15360.3 (PMC10907879; doi:10.12688/openreseurope.15360.3)
Supplement: Peer review history

# Reviewer report: Reviewer response for version 3

**DOI:** 10.21956/openreseurope.18591.r38144

**Published:** 2024-03-01

**Authors:** Juan B. Calanche M

**Affiliations:** 1 Universidad de Zaragoza-CITA, Miguel Servet 177, 50013 Zaragoza,, Spain

No comments.

If applicable, is the statistical analysis and its interpretation appropriate?

Partly

Is the study design appropriate and is the work technically sound?

Yes

Is the work clearly and accurately presented and does it engage with the current literature?

Yes

Are the conclusions drawn adequately supported by the results?

Partly

Are sufficient details of methods and analysis provided to allow replication by others?

Partly

Are all the source data and materials underlying the results available?

Yes

Reviewer Expertise:

Food development and Sensory analysis. (Meat and fish science and technology).

I confirm that I have read this submission and believe that I have an appropriate level of expertise to confirm that it is of an acceptable scientific standard.

# Reviewer report: Reviewer response for version 3

**DOI:** 10.21956/openreseurope.18591.r38143

**Published:** 2024-02-16

**Authors:** Kaiser Younis

**Affiliations:** 1 Department of Food Technology, Islamic University of Science and Technology, Pulwama, Jammu and Kashmir, India

I have no further comments to make.

If applicable, is the statistical analysis and its interpretation appropriate?

Yes

Is the study design appropriate and is the work technically sound?

Yes

Is the work clearly and accurately presented and does it engage with the current literature?

Yes

Are the conclusions drawn adequately supported by the results?

Yes

Are sufficient details of methods and analysis provided to allow replication by others?

Yes

Are all the source data and materials underlying the results available?

Yes

Reviewer Expertise:

My area of expertise is food product development.

I confirm that I have read this submission and believe that I have an appropriate level of expertise to confirm that it is of an acceptable scientific standard.

# Reviewer report: Reviewer response for version 2

**DOI:** 10.21956/openreseurope.17630.r36333

**Published:** 2024-01-22

**Authors:** Juan B. Calanche M

**Affiliations:** 1 Universidad de Zaragoza-CITA, Miguel Servet 177, 50013 Zaragoza,, Spain

This article offers a compelling exploration into the intricacies of constructing a semantic lexicon for the sensory characterization of plant-based meat substitute products. The comprehensive methodology employed encompasses diverse approaches, including focus groups, hedonic testing, descriptor identification and selection, quantitative descriptive analysis, and performance evaluation. While the study's breadth and ambition are noteworthy, its substantial findings make a significant contribution to the research landscape in this domain. However, to enhance the scientific rigour of the work, certain sections may benefit from refinement, clarification, or the incorporation of additional information. The ensuing breakdown provides a detailed analysis of specific comments aimed at optimizing the scientific quality of the manuscript.

 1) **Plain language summary:** The phrase "but rather their salvation" is questioned for its appropriateness, as the concept of salvation lacks clarity. Further clarification is needed to convey a more precise message to consumers.

 2) **Methods (Ingredient):** Elaboration on the rationale behind ingredient selection and a clarification of the criteria governing proportion choices in the formulations would enhance methodological transparency.

 3) **Article:** Provide an early explanation of the acronym "AZTI" within the article for reader comprehension.

 4) **Methods (Demographic characteristics):** Clarify the purpose of the demographic characteristics section, particularly regarding the necessity of gender balance among sensory panel members. Additionally, justify the predetermined characteristics of the selected sample, considering its relatively small size.

 5) **Methods. Sensory analysis:** Identify the entity endorsing the ethical certification of the experiment following ISO 8586:2012, specifying the relevant paragraph or section.

 6) **Methods. Recruitment of judges and basic training: **Provide the rationale for excluding non-consumers of plant-based products and elucidate the criteria used to establish these selection criteria.

 7) **Methods. Recruitment of judge: **Justify the choice of 8 assessors and address the discrepancy with ISO 8586:2023 (4.2.5 Number of persons to be selected), which recommends a minimum of 12 trained assessors for a sensory panel.

 8) **Methods: **Explain the omission of an independent hedonic analysis to determine the product with the highest acceptability. Additionally, clarify the rationale behind using the same candidates for both hedonic and quantitative descriptive tests.

 9) **Methods. Generation of descriptors and training of judges:** Specify how the sauce's impact on sensory perception was accounted for during the selection of organoleptic attributes for the vegan meatball. Clarify the considerations given to the sauce-meat sausage interaction.

 10) **Methods. Generation of descriptors and training of judges:** Justify the choice of not using typical meatballs as reference products for imitation when substituting a meat product with a plant-based alternative. Discuss the potential insights gained from comparing the profiles of the original food with its various analogues.

 11) **Methods. T Generation of descriptors and training of judges: **Provide details on the process of sharing, selecting, and standardizing the final organoleptic attributes after obtaining general descriptors from participants during training. Clarify the methodology employed in evaluating the plant-based product.

 12) **Methods. T Generation of descriptors and training of judges:** Consider replacing the term "panellist" with sensory, screened, or trained sensory assessor, as deemed appropriate, to align with the evolution of assessors during training, as per ISO 8586:2023 standards.

 13) **Results and discussion.** Glossary of descriptors for plant-based meatballs: Address the conceptual inconsistency in defining "characteristic taste" as the flavour of meat, given the distinct sensory receptors for taste and flavour. According to ISO 5492:2008 the term “taste” only corresponds to the basic ones (acid, salty, sweet, bitter and umami) and can include alkaline and metallic. For that reason, must clarify the definitions of "flavour" and "taste" to enhance understanding, particularly concerning the perceived difficulty in assessing this organoleptic property.

 14) **Results and discussion.** Sensory profile of ready-to-eat plant-based “meatballs” Fig. 6: Clearly state whether the observed differences between the attributes of the two types of burgers are statistically significant, providing additional information on the statistical analysis employed.

 15) **Results and discussion:** Provide insights into how outliers and deviations were addressed during training, including specific actions taken in response to individual tasters' performance. This information is crucial for enhancing the robustness of future research or training endeavours.

 16) **Conclusions:** The conclusions of this study should be revisited and refined in consideration of the aforementioned observations. Incorporating these insights will contribute to a more robust and nuanced interpretation of the findings, ensuring the overall integrity and scientific merit of the article.

If applicable, is the statistical analysis and its interpretation appropriate?

Partly

Is the study design appropriate and is the work technically sound?

Yes

Is the work clearly and accurately presented and does it engage with the current literature?

Yes

Are the conclusions drawn adequately supported by the results?

Partly

Are sufficient details of methods and analysis provided to allow replication by others?

Partly

Are all the source data and materials underlying the results available?

Yes

Reviewer Expertise:

Food development and Sensory analysis. (Meat and fish science and technology).

I confirm that I have read this submission and believe that I have an appropriate level of expertise to confirm that it is of an acceptable scientific standard, however I have significant reservations, as outlined above.

## References

[1] : Innovative Plant-Based Burger Enriched with Tenebrio molitor Meal: Characterization and Shelf-Life. *Foods* .2023;12(18) : 10.3390/foods12183460 10.3390/foods12183460 37761169 PMC10528019

# Response

**Published:** 2024-01-24

**Authors:** Clara Talens

**Affiliations:** AZTI, Food Research, Basque Research and Technology Alliance (BRTA), Spain

In response to the insightful feedback received from the reviewers, we have systematically addressed each comment to ensure that our study on the sensory is presented with greater precision and depth: 1)           Plain language summary: The phrase "but rather their salvation" is questioned for its appropriateness, as the concept of salvation lacks clarity. Further clarification is needed to convey a more precise message to consumers. The phrase "but rather their salvation" has been modified to provide more clarity: "Imagine a future generation of children who, from a young age, have learned that vegetables are not just an alternative to meat, but a wholesome choice for nutrition and health." 2)           Methods (Ingredient): Elaboration on the rationale behind ingredient selection and a clarification of the criteria governing proportion choices in the formulations would enhance methodological transparency. We agree that providing criteria for ingredient selection is important. In our study, the choice of lentils, quinoa, and oats was based on their nutritional properties, availability, and compatibility with the target product's sensory attributes. We aimed to create a plant-based meatball that is both nutritionally balanced and appealing to consumers. However, we acknowledge the need for detailed information on the nutritional aspects of the chosen ingredients to prevent any deficiencies. We have included a paragraph in the Materials and methods section. 3)           Article: Provide an early explanation of the acronym "AZTI" within the article for reader comprehension. AZTI is not an acronym but the name of the institution where the flour was processed, location is included. AZTI is one of the affiliations of the manuscript. 4)           Methods (Demographic characteristics): Clarify the purpose of the demographic characteristics section, particularly regarding the necessity of gender balance among sensory panel members. Additionally, justify the predetermined characteristics of the selected sample, considering its relatively small size. The necessity of gender balance among sensory panel members is crucial for ensuring diverse sensory perceptions. To clarify, we can add: "Gender balance was aimed to gather a wide range of sensory feedback, reflecting a varied panellist base." 5)           Methods. Sensory analysis: Identify the entity endorsing the ethical certification of the experiment following ISO 8586:2012, specifying the relevant paragraph or section. This was already specified in the sentence: “The ethical approval was conducted according to quality standards of the ISO 8586:2012 certified by AENOR, the Spanish Association for Standardization and Certification.” 6)           Methods. Recruitment of judges and basic training: Provide the rationale for excluding non-consumers of plant-based products and elucidate the criteria used to establish these selection criteria. The rationale for excluding non-consumers of plant-based products is based on targeting a specific consumer group. We can clarify by stating: "Non-consumers were excluded to focus on feedback from potential regular consumers of plant-based products." 7)           Methods. Recruitment of judge: Justify the choice of 8 assessors and address the discrepancy with ISO 8586:2023 (4.2.5 Number of persons to be selected), which recommends a minimum of 12 trained assessors for a sensory panel. The following sentence was added: Eight assessors were chosen based on the availability and resources, acknowledging the deviation from ISO 8586:2023's recommendation of a minimum of 12. 8)           Methods: Explain the omission of an independent hedonic analysis to determine the product with the highest acceptability. Additionally, clarify the rationale behind using the same candidates for both hedonic and quantitative descriptive tests. The main objective of the study was to train judges for sensory analysis, rather than conducting hedonic tests. Throughout these sessions, a combination of methodologies was employed to generate descriptors, and a structured approach was followed to train the judges. The training encompassed discussions and consensus-building exercises to ensure a shared understanding of the sensory characteristics of the plant-based meatballs. 9)           Methods. Generation of descriptors and training of judges: Specify how the sauce's impact on sensory perception was accounted for during the selection of organoleptic attributes for the vegan meatball. Clarify the considerations given to the sauce-meat sausage interaction. We added the following paragraph: “In the sensory analysis of plant-based meatballs, a critical step involved the removal of the sauce to focus solely on the meatball's inherent sensory properties. This was achieved through a careful and standardized process using a napkin. Each meatball, initially prepared with sauce, was gently lifted from its sauce. Then, using a napkin, the surface of the meatball was dabbed to absorb the sauce. This dabbing was performed with enough care to ensure that the sauce was absorbed without exerting pressure that might alter the texture of the meatball. The goal was to remove any visible traces of the sauce, effectively isolating the meatball from external flavor influences. This method ensured that each meatball was treated uniformly, allowing for an accurate and focused assessment of its intrinsic sensory characteristics.” 10)        Methods. Generation of descriptors and training of judges: Justify the choice of not using typical meatballs as reference products for imitation when substituting a meat product with a plant-based alternative. Discuss the potential insights gained from comparing the profiles of the original food with its various analogues. We added the following sentence: “The focus was on developing a distinct plant-based product rather than directly imitating meat-based meatballs." 11)        Methods. T Generation of descriptors and training of judges: Provide details on the process of sharing, selecting, and standardizing the final organoleptic attributes after obtaining general descriptors from participants during training. Clarify the methodology employed in evaluating the plant-based product. Regarding the process of sharing, selecting, and standardizing the final organoleptic attributes, we would like to clarify that the details of this methodology have been comprehensively provided in the manuscript. However, acknowledging the importance of ease of understanding for the reader, we have incorporated additional connectors and transitional phrases within the text. 12)        Methods. T Generation of descriptors and training of judges: Consider replacing the term "panellist" with sensory, screened, or trained sensory assessor, as deemed appropriate, to align with the evolution of assessors during training, as per ISO 8586:2023 standards. Agree. The term 'panellist' has been replaced with 'trained sensory assessor' to better reflect their role. 13)        Results and discussion. Glossary of descriptors for plant-based meatballs: Address the conceptual inconsistency in defining "characteristic taste" as the flavour of meat, given the distinct sensory receptors for taste and flavour. According to ISO 5492:2008 the term “taste” only corresponds to the basic ones (acid, salty, sweet, bitter and umami) and can include alkaline and metallic. For that reason, must clarify the definitions of "flavour" and "taste" to enhance understanding, particularly concerning the perceived difficulty in assessing this organoleptic property. We sincerely appreciate the reviewer's insightful observation on this matter, as it significantly enhances the depth and clarity of our study. The term 'taste' is clarified to represent the basic taste sensations, and 'flavour' is used to describe the overall sensory experience. We have included the following explanation. *In English, the terms "taste" and "flavor" have distinct meanings, which can lead to confusion in translation, especially into Spanish.* *Taste: This refers to the sensation produced when a substance in the mouth reacts chemically with taste receptor cells located on taste buds. Taste is limited to five basic qualities: sweet, sour, salty, bitter, and umami. These basic tastes are perceived through the tongue.* *Flavour: It is a broader concept that includes not only the basic tastes but also the aromas and other sensations (like spiciness or temperature) perceived through the olfactory receptors in the nose. Flavour is the overall perception of a food or drink, combining taste, smell, and other sensory attributes.* *When translating these terms into Spanish, the distinction can become blurred:* *"Taste" is translated to “gusto” or “sabor.” “Gusto” aligns more with the English "taste," referring to the basic taste sensations. However, “sabor” can be interpreted as both “taste” and “flavour,” encompassing the broader sensory experience.* *"Flavour" is often translated as “sabor,” but this doesn't fully capture the complexity of the term as understood in English, which includes aroma and other sensory experiences beyond just taste.* *The confusion arises because “sabor” in Spanish can mean both the basic tastes perceived by the tongue and the overall flavour experience, which includes aroma and other factors. This overlap in meaning can make it challenging to convey the distinct concepts of "taste" and "flavour" from English into Spanish accurately.* *In the context of this study conducted in Spain, the term "taste" (or "gusto" in Spanish) can often be understood to encompass what is referred to as "overall flavour" in English. This broader interpretation in the Spanish context arises from linguistic and cultural nuances in how sensory experiences are described. Therefore, in this study, when the term "taste" is used, especially given its Spanish context, it is aligned more with the English concept of "overall flavour." This encompasses a more holistic sensory assessment rather than just the basic taste sensations. Such linguistic and cultural differences are important to consider in sensory studies, as they can influence how sensory data is described, interpreted, and communicated.* 14)        Results and discussion. Sensory profile of ready-to-eat plant-based “meatballs” Fig. 6: Clearly state whether the observed differences between the attributes of the two types of burgers are statistically significant, providing additional information on the statistical analysis employed. The statistical significance (p value) of the difference between the two samples is shown in Table 5. We have included additional information near the description of Figure 6 to clarify the statistical analysis and findings, enhancing the reader's understanding of the presented data. 15)        Results and discussion: Provide insights into how outliers and deviations were addressed during training, including specific actions taken in response to individual tasters' performance. This information is crucial for enhancing the robustness of future research or training endeavours. In response to the reviewer's comment about addressing outliers and deviations during training, as well as actions taken in response to individual tasters' performance, the study does provide detailed information on these aspects. The training of judges was performed in compliance with international standards, involving thirteen 1-hour sessions over three months, covering the selection of commercial reference, generation of descriptors, training of the panel, and validation of the trained panel and product characterization​​. Specific actions taken in response to individual tasters' performance included scrutinizing the descriptors and judges whose standard deviation was ≥ 1 after each tasting session. It was ensured that the difference between sessions was not significant at the 0.05 level, otherwise, it indicated that the scores of individual tasters were inconsistent​​. Additional three sessions were conducted to re-train the tasters for descriptors with high variability. A particular emphasis was placed on the definition and intensity of these descriptors​. The study illustrates a rigorous approach to ensuring the consistency and reliability of the panel's evaluations. The panel's performance was deemed repeatable and reproducible, and an adequate capacity for discrimination was determined before commencing the sensory evaluation​​. The study also reports the statistical analyses employed, including a one-way ANOVA conducted per taster at the end of each session to identify statistically significant differences, and a three-way ANOVA to analyse the significance of the taster, descriptor, and session​​. It is important to note that further details on the analysis conducted can be found in the “Underlying data” section of the study. 16)        Conclusions: The conclusions of this study should be revisited and refined in consideration of the aforementioned observations. Incorporating these insights will contribute to a more robust and nuanced interpretation of the findings, ensuring the overall integrity and scientific merit of the article. In light of the valuable feedback from our reviewers, our study's conclusions have been refined.

# Reviewer report: Reviewer response for version 2

**DOI:** 10.21956/openreseurope.17630.r33730

**Published:** 2023-07-17

**Authors:** Kaiser Younis

**Affiliations:** 1 Department of Food Technology, Islamic University of Science and Technology, Pulwama, Jammu and Kashmir, India

All the queries have been addressed by the authors.

If applicable, is the statistical analysis and its interpretation appropriate?

Yes

Is the study design appropriate and is the work technically sound?

Yes

Is the work clearly and accurately presented and does it engage with the current literature?

Yes

Are the conclusions drawn adequately supported by the results?

Yes

Are sufficient details of methods and analysis provided to allow replication by others?

Yes

Are all the source data and materials underlying the results available?

Yes

Reviewer Expertise:

My area of expertise is food product development.

I confirm that I have read this submission and believe that I have an appropriate level of expertise to confirm that it is of an acceptable scientific standard.

# Reviewer report: Reviewer response for version 1

**DOI:** 10.21956/openreseurope.16605.r33405

**Published:** 2023-07-03

**Authors:** Kaiser Younis

**Affiliations:** 1 Department of Food Technology, Islamic University of Science and Technology, Pulwama, Jammu and Kashmir, India

The authors have done a commendable job of training a sensory panel and formulating a glossary of descriptors for plant-based meatballs. Their efforts are likely to be of great benefit to researchers who conduct sensory analysis on similar products. However, there are a few areas where the research could be improved. The following suggestions are provided:

1. Please indicate the criteria for choosing the ingredients, as plant-based meat is recommended for various reasons but requires a sophisticated knowledge of plant foods to prevent deficiencies due to low-quality or deficient amino acids.
2. I was wondering why the meatballs haven't been tested for different flavors and textures. I think it would be helpful to determine what types of meatballs are most appealing to consumers. Do you have any plans to do this in the future?
3. The core temperature of the product during the process must be specified in the paper to ensure that the meatballs are cooked to a safe temperature and are safe to eat.
4. The type of light in the sensory rooms can significantly affect the results of sensory analysis. Please indicate the source of light in your report.
5. The research could have considered offering remuneration to the participants. This might have encouraged more people to apply and could have resulted in a more diverse group of participants. The paper does not inform about the reason of taking only 8 panelists.
6. The paper could have also considered using a different reference product for the hedonic evaluation. This might have given the judges a different perspective on the products being evaluated.
7. The paper could have considered providing more detail about the training sessions. For example, they could have described the specific exercises that the judges were asked to perform.
8. The paper could have benefited from providing more information about the outliers in the data. This could have included explaining why the outliers occurred and whether they were considered to be significant.
9. The paper could have included more participants in the study. This would help to increase the generalizability of the results. For example, the researchers could have recruited participants from a variety of demographics, such as age, gender, and dietary preferences.
10. The paper could have been strengthened by including a control group in the study. This would have allowed the researchers to compare the sensory profile of the two products to a baseline, such as a group of participants who were not familiar with plant-based "meatballs."
11. A follow-up study could have been conducted to assess the long-term sensory properties of the two products, which would have helped to determine whether the sensory profile of the products changed over time.
12. The paper could benefit from a section that discusses the implications of the study for future research. For example, the authors could discuss the possibility of using the glossary of descriptors to develop a sensory profile for other plant-based products.

If applicable, is the statistical analysis and its interpretation appropriate?

Yes

Is the study design appropriate and is the work technically sound?

Yes

Is the work clearly and accurately presented and does it engage with the current literature?

Yes

Are the conclusions drawn adequately supported by the results?

Yes

Are sufficient details of methods and analysis provided to allow replication by others?

Yes

Are all the source data and materials underlying the results available?

Yes

Reviewer Expertise:

My area of expertise is food product development.

I confirm that I have read this submission and believe that I have an appropriate level of expertise to confirm that it is of an acceptable scientific standard, however I have significant reservations, as outlined above.

# Response

**Published:** 2023-07-09

**Authors:** Clara Talens

**Affiliations:** AZTI, Food Research, Basque Research and Technology Alliance (BRTA), Spain

Thank you for your valuable feedback and suggestions regarding our research on sensory analysis of plant-based meatballs. We appreciate your positive comments and acknowledge the areas where the study could be further improved. We will address each of your suggestions below:

 1. Criteria for choosing ingredients: We agree that providing criteria for ingredient selection is important. In our study, the choice of lentils, quinoa, and oats was based on their nutritional properties, availability, and compatibility with the target product's sensory attributes. We aimed to create a plant-based meatball that is both nutritionally balanced and appealing to consumers. However, we acknowledge the need for detailed information on the nutritional aspects of the chosen ingredients to prevent any deficiencies. We have included a paragraph in the Materials and methods section.

 2. Testing different flavors and textures: We recognize the importance of investigating a wider range of flavors and textures to gain insights into consumer preferences. However, in this study, our primary focus was on developing a sensory lexicon for the base product as a foundational step. By establishing a robust lexicon and ensuring panel agreement, we can subsequently utilize it to explore and evaluate a broader variety of flavors and textures in future research, thereby addressing the valuable suggestion put forth by the reviewer

 3. Core temperature during processing: We would like to assure you that the paper already includes the information you suggested. Specifically, we stated in the manuscript that "The temperature at the core of the product was maintained at 105—108 °C for 34 min, achieving an F 0 of 3, then the samples were stored at ambient temperature." We apologize if this information was not immediately evident, and we will ensure that it is presented more prominently for clarity in future revisions.

 4. Sensory room lighting: We appreciate your point regarding the potential influence of lighting conditions on sensory analysis. In our study, we conducted sensory evaluations in controlled environments with standardized lighting conditions. We have included this information “The sensory rooms were equipped with full-spectrum daylight LED lighting, which ensures consistent illumination across evaluations”.

 5. Participant remuneration and panel size: We acknowledge that offering remuneration to participants could have increased the diversity of our panel. While we didn't provide monetary compensation to the trined panellists in this study, we ensured their commitment and motivation by providing thorough training and a supportive environment. As for the panel size, we aimed for a manageable group that allows for effective training and panel performance. There are indeed a significant amount of research with such number of trained panellist.

 6. Reference product for hedonic evaluation: Selecting an appropriate reference product is indeed critical in sensory evaluations, as it provides a benchmark for comparison and helps assess the relative attributes of the tested products. In our study, the primary objective was to enhance the sensory profile of plant-based meatballs in sauce, specifically focusing on the development of a lexicon to evaluate their characteristics. The chosen reference product represented the existing market offering in terms of plant-based meatballs in sauce that are shelf-stable. Introducing other types of reference products could have deviated from the main aim of improving the existing options available to consumers. We have included a paragraph to explain this decision.

 7. Detailed training sessions: We have included a more detailed description of these exercises in the revised paper to enhance transparency and reproducibility.

 8. Outliers in the data: Thank you for highlighting the need for addressing outliers in the data and providing explanations for their occurrence. We agree that discussing the outliers would contribute to a better understanding of the sensory profile and potential variations within the panel. In our future work, we will thoroughly analyze and document outliers, including their significance and potential causes. The presence of outliers in the data is an essential aspect to consider when analyzing sensory profiles. In the study, Figure 2 and Figure 4 provide visual representations of the data using boxplots, where the black dots represent the outliers. These outliers signify data points that deviate significantly from the majority of the observed values. While the paper did not explicitly explain the reasons behind the occurrence of outliers, they can arise due to various factors, such as individual differences in sensory perception, variations in panellists' responses, or potential measurement errors. It is important to note that outliers may or may not have a significant impact on the overall analysis, and their interpretation should be approached cautiously. In this study, the focus was primarily on assessing the variability and robustness of the panel over multiple sessions. The reduced variability observed in Table 4 and the alignment of the red squares (representing mean values) in Figure 4 indicate improved panel consensus and reduced variability for descriptors such as cohesiveness, juiciness, and taste after the second training. This suggests that the outliers may not have exerted a significant influence on the overall analysis or affected the interpretation of the sensory profiles. It is worth mentioning that the sensory profiles of the prototype samples (depicted in Figure 5) fell within the acceptable range defined by the reference sample's agreed-upon QDA area for descriptors like firmness, fragility, inner colour, outer colour, and chewiness. While the presence of outliers can provide valuable insights, their impact on the study findings may be relatively minimal in this context.

 9. Increased participant diversity: We appreciate the reviewer's comment regarding the inclusion of more participants in the study to enhance the generalizability of the results. In this research, the focus was on training a panel of sensory experts to assess plant-based meat analogues. Thus, the recruitment process prioritized individuals who were consumers of this type of product to ensure their familiarity with the sensory characteristics and attributes of plant-based meatballs. Additionally, efforts were made to maintain a gender balance among the panellists to minimize potential gender-related biases. While variables such as demographics and age are relevant in consumer studies, they carry less significance in the context of training sessions for sensory panels. The primary objective of the training sessions was to develop a panel of trained assessors with the ability to detect and discriminate flavour and texture differences in the plant-based meatball products accurately. As such, the selection process focused on individuals who demonstrated the sensory acuity and aptitude necessary for trained panel evaluations, irrespective of their specific demographics or age. It is important to note that the expertise and ability to discern subtle sensory differences are paramount for panellists involved in the development of a sensory lexicon. Thus, the focus was on recruiting panellists who could contribute to the development of a reliable and consistent sensory evaluation process. While expanding the participant pool to encompass a broader range of demographics could be beneficial for consumer studies, it was not the primary aim of this study, which focused on training and validating a specific panel for sensory analysis. Future research could certainly explore the impact of demographic variables on consumer acceptance and preferences, and we acknowledge the potential value of conducting such studies.

 10. Control group inclusion: We appreciate the reviewer's suggestion regarding the inclusion of a control group in the study to compare the sensory profiles of the plant-based meatballs with a baseline group unfamiliar with such products. However, it is important to note the fundamental difference between consumer studies and trained panel evaluations. In this study, the focus was on training and validating a sensory panel with the objective of obtaining objective and quantified measurements of sensory attributes for the plant-based meatball products. Trained panels are specifically trained to evaluate and quantify sensory characteristics using standardized methodologies, resulting in measures with the input of trained human assessors. The emphasis is on capturing detailed sensory profiles and discriminating specific attributes, rather than assessing overall acceptance or rejection. On the other hand, consumer studies aim to investigate the acceptance, preferences, and overall liking of food products among a target population. The inclusion of a control group in consumer studies can provide a valuable baseline for comparison, as it allows for a comparison between a familiar product and a novel or alternative product in terms of overall acceptance. In the context of a trained panel study, the focus is on obtaining precise and reliable sensory measurements rather than capturing broad acceptance or preference data. The trained panel's expertise lies in quantifying sensory attributes and providing detailed insights into the specific sensory characteristics of the plant-based meatballs. While the inclusion of a control group could be valuable for assessing consumer acceptance, it may not align with the objective of the present study, which aimed to develop a sensory lexicon and obtain quantified sensory data. However, future research could certainly explore the incorporation of consumer studies to complement the findings from trained panel evaluations and provide a more comprehensive understanding of the sensory profiles and acceptance of plant-based meat products.

 11. Long-term sensory properties: We agree that such an investigation would provide valuable insights into the shelf-life stability of the products. After the submission of the article, we conducted a shelf-life assessment of the plant-based meatballs at the three-month time point. The results confirmed the sensory stability of the products up to that duration. However, the study is still ongoing, and assessments at the six-month, nine-month, and twelve-month time points are planned to comprehensively evaluate the long-term sensory properties and potential changes that may occur over an extended period. By conducting these additional assessments, we aim to gain a better understanding of the shelf-life stability and sensory characteristics of the plant-based meatball products over an extended timeframe. The data obtained from these subsequent evaluations will contribute to the overall understanding of the products' sensory profiles and guide recommendations for storage conditions and product shelf-life.

 12. Implications for future research: We agree that highlighting the potential applications and future directions of our findings would be valuable for advancing the field of sensory analysis for plant-based products. One important avenue for future research is the continued assessment of the long-term sensory properties and shelf-life stability of the plant-based meatball products beyond the three-month timeframe covered in this study. Conducting additional evaluations at six-month, nine-month, and twelve-month intervals would provide further insights into the changes in sensory characteristics over time and help establish appropriate storage conditions to maintain product quality. Furthermore, the developed lexicon of descriptors can serve as a valuable resource for future sensory evaluations of other plant-based products. However, it is important to note that the descriptors generated in this study were tailored specifically to the sensory profile of the plant-based meatballs in sauce. If applied to different product categories, it would be necessary to generate a new lexicon tailored to those specific products. Nevertheless, the benefit of having a trained panel, as established in this study, is that the panellists have already undergone extensive training and are familiar with the various steps involved in sensory evaluation. This reduces the training time required for future studies and ensures the availability of a reliable and consistent panel for assessing different plant-based products. We had added a paragraph for future work in the new submission. Thank you again for your thoughtful comments and suggestions. Your feedback will greatly contribute to enhancing the quality and comprehensiveness of our research.
